# Chest magnetic resonance imaging in cystic fibrosis: technique and clinical benefits

**DOI:** 10.1007/s00247-022-05539-9

**Published:** 2022-11-14

**Authors:** Daniel Gräfe, Freerk Prenzel, Franz Wolfgang Hirsch

**Affiliations:** 1 Department of Pediatric Radiology, Leipzig University Hospital, Liebigstraße 20a, 04103 Leipzig, Germany; 2 Department of Pediatrics, Leipzig University Hospital, Liebigstraße 20a, 04103 Leipzig, Germany

**Keywords:** Bronchiectasis, Chest, Children, Computed tomography, Cystic fibrosis, Eichinger score, Fourier decomposition, Magnetic resonance imaging, Pulmonary, Ultra-short echo times

## Abstract

Cystic fibrosis (CF) is one of the most common inherited and life-shortening pulmonary diseases in the Caucasian population. With the widespread introduction of newborn screening and the development of modulator therapy, tremendous advances have been made in recent years both in diagnosis and therapy. Since paediatric CF patients tend to be younger and have lower morbidity, the type of imaging modality that should be used to monitor the disease is often debated. Computed tomography (CT) is sensitive to many pulmonary pathologies, but radiation exposure limits its use, especially in children and adolescents. Conventional pulmonary magnetic resonance imaging (MRI) is a valid alternative to CT and, in most cases, provides sufficient information to guide treatment. Given the expected widespread availability of sequences with ultra-short echo times, there will be even fewer reasons to perform CT for follow-up of patients with CF. This review aims to provide an overview of the process and results of monitoring CF with MRI, particularly for centres not specialising in the disease.

## Introduction

Cystic fibrosis (CF) is one of the most common, inherited and life-limiting diseases in the Caucasian population. The mean prevalence in European Union (EU) countries is 0.74/10,000, with an incidence of 1/3,000 to 1/6,000 live births [1, 2]. A gene defect encoding a chloride channel causes this multisystemic disease whereby progressive lung disease is the leading cause of death [3]. While in the past CF often resulted in demise in childhood and adolescence, improvements in therapy and diagnostics have increased the median life expectancy into mid-adulthood [4]. Two factors have significantly altered the management of CF and, thus, survival in recent years: the widespread introduction of newborn screening, resulting in presymptomatic therapy [5] and the development of modulator therapies, allowing treatment of the defective chloride channel [6].

## Why image the lungs?

Physical examination, microbiological and pulmonary function tests and laboratory parameters are used to monitor the progression of the disease. Structural lung changes occur in infancy and during the preschool period and may be missed in asymptomatic children [7, 8]. However, high-resolution cross-sectional imaging can detect abnormalities in the lungs more sensitively than lung function diagnostics and, thus, direct therapy at an early stage [8, 9]. It has been shown that in infants and young children, magnetic resonance imaging (MRI) correlates well with the (sensitive) lung clearance index [10]. In addition, imaging can sometimes identify the cause of pulmonary deterioration or even distinguish between fungal and other microbial causes of infection, enabling targeted therapy [11].

## What do clinicians need to know?

Ideally, the treating pulmonologist needs precise and comprehensible parameters by which to assess the progression of the disease and from which they can immediately derive clues for patient management. The imaging modality is not the primary objective for the clinician. The radiologist, on the other hand, must be aware of the perspective and requests of the referring physician, even if they have not been explicitly phrased. Before any examination, consider:

a) Are there changes over time that have prognostic or therapeutic relevance for the patient? Multiple scores for each imaging modality are useful for such quantification of longitudinal trends [12–15].

b) Is there an acute event that requires immediate therapy? Examples include acute respiratory tract infective exacerbations or pulmonary haemorrhage. The radiologic assessment should, irrespective of the score, explicitly evaluate and report this aspect.

**Fig. 1:**
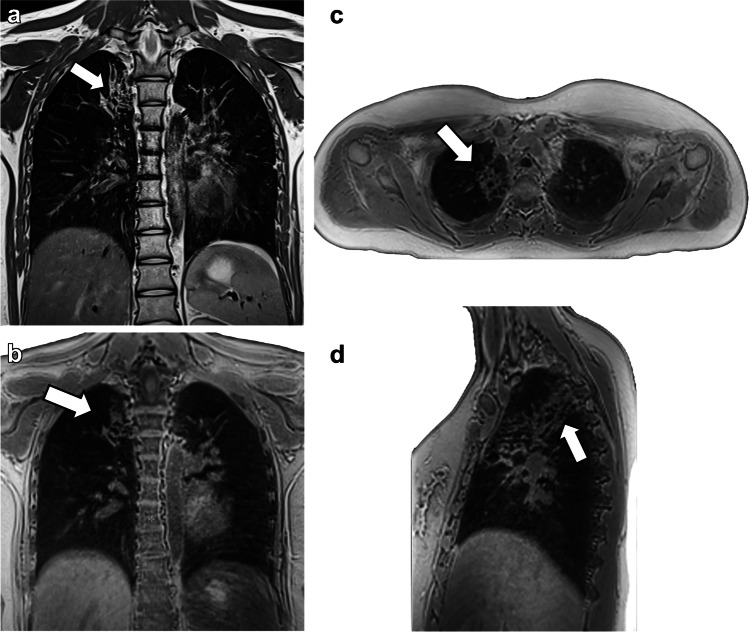
Magnetic resonance images in a 16-year-old girl with bronchiectasis in the upper lobes (*arrows*). **a** Coronal respiratory-triggered T2 fast spin echo and self-gated ultra-short echo time sequences with (**b**) coronal, (**c**) axial and (**d**) sagittal multiplanar reconstructions. Please note that air trapping distal to the dilated bronchi is only depicted in the latter

**Fig. 2:**
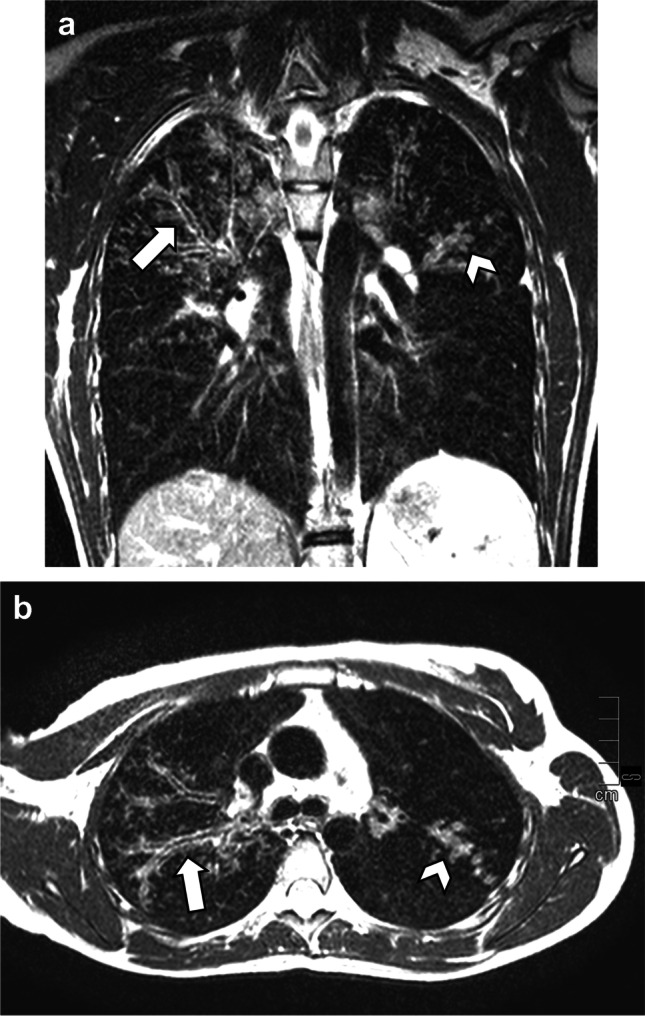
Coronal (**a**) and axial (**b**) T2 fast spin echo images in a 16-year-old girl with severe cystic fibrosis and infection with *Staphylococcus aureus* and *Pseudomonas aeruginosa* show signs of bronchiectasis in the right upper lobe (*arrows*) and small mucus plugs in the left upper lobe (*arrowheads*)

**Fig. 3:**
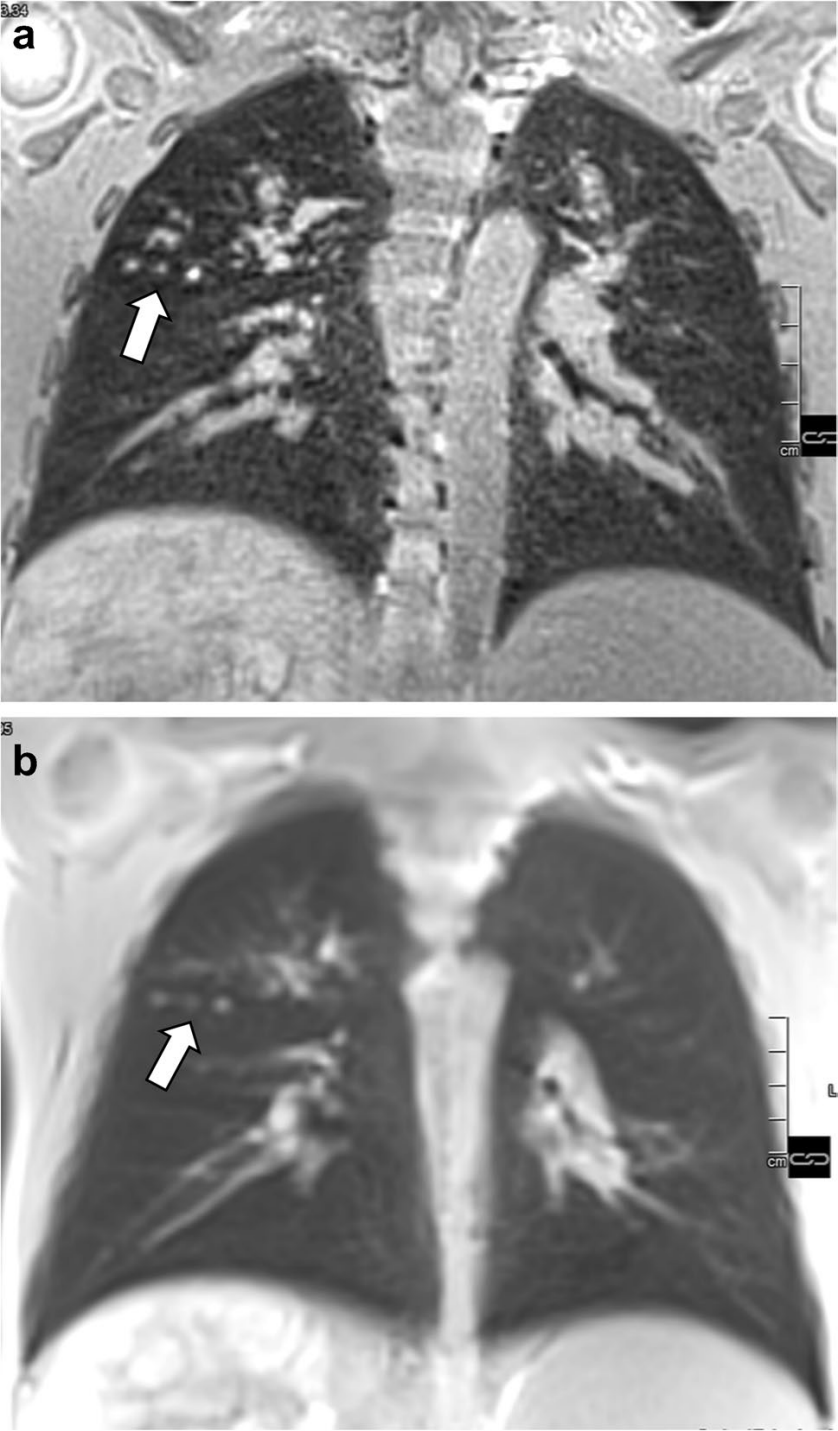
A coronal ultra-short echo time sequence in a 10-year-old boy with cystic fibrosis and good pulmonary function. Self-gating (**a**) with an acquisition time of 6:15 min. Single breath-hold technique (**b**) with an acquisition time of 17 s. Peripheral mucus is found in both upper lobes (*arrows*)

**Fig. 4:**
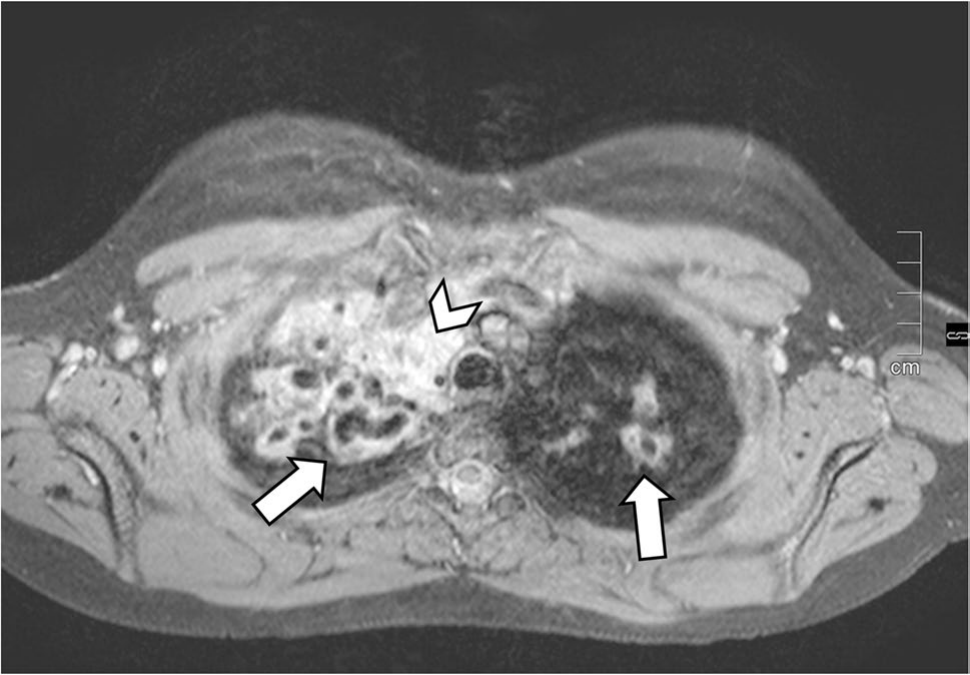
An axial T2 fast spin echo image with fat saturation in a 12-year-old girl with severe pulmonary involvement in cystic fibrosis. Marked bronchiectasis and bronchial wall thickening affect both upper lobes (*arrows*). In addition, minor consolidations are seen in the right upper lobe (*arrowhead*)

**Fig. 5:**
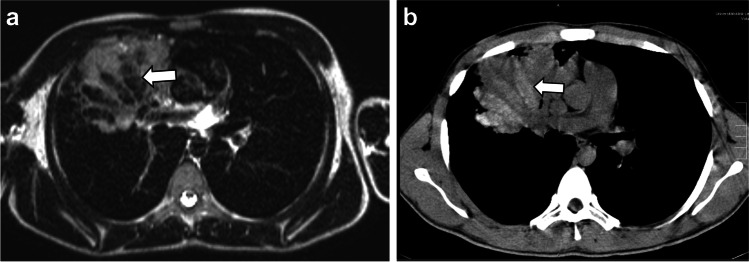
A 16-year-old boy with cystic fibrosis. A T2 fast spin echo magnetic resonance (MR) image (**a**). The mucus in the bronchiectasis in the basal right upper lobe is hypointense on T2 (*arrow*). An axial unenhanced computed tomography (CT) image (**b**). The mucus in the bronchiectasis in the basal right upper lobe is hyperdense on CT (*arrow*). This combination of MR and CT findings is characteristic of allergic bronchopulmonary aspergillosis and aspergilloma

**Fig. 6:**
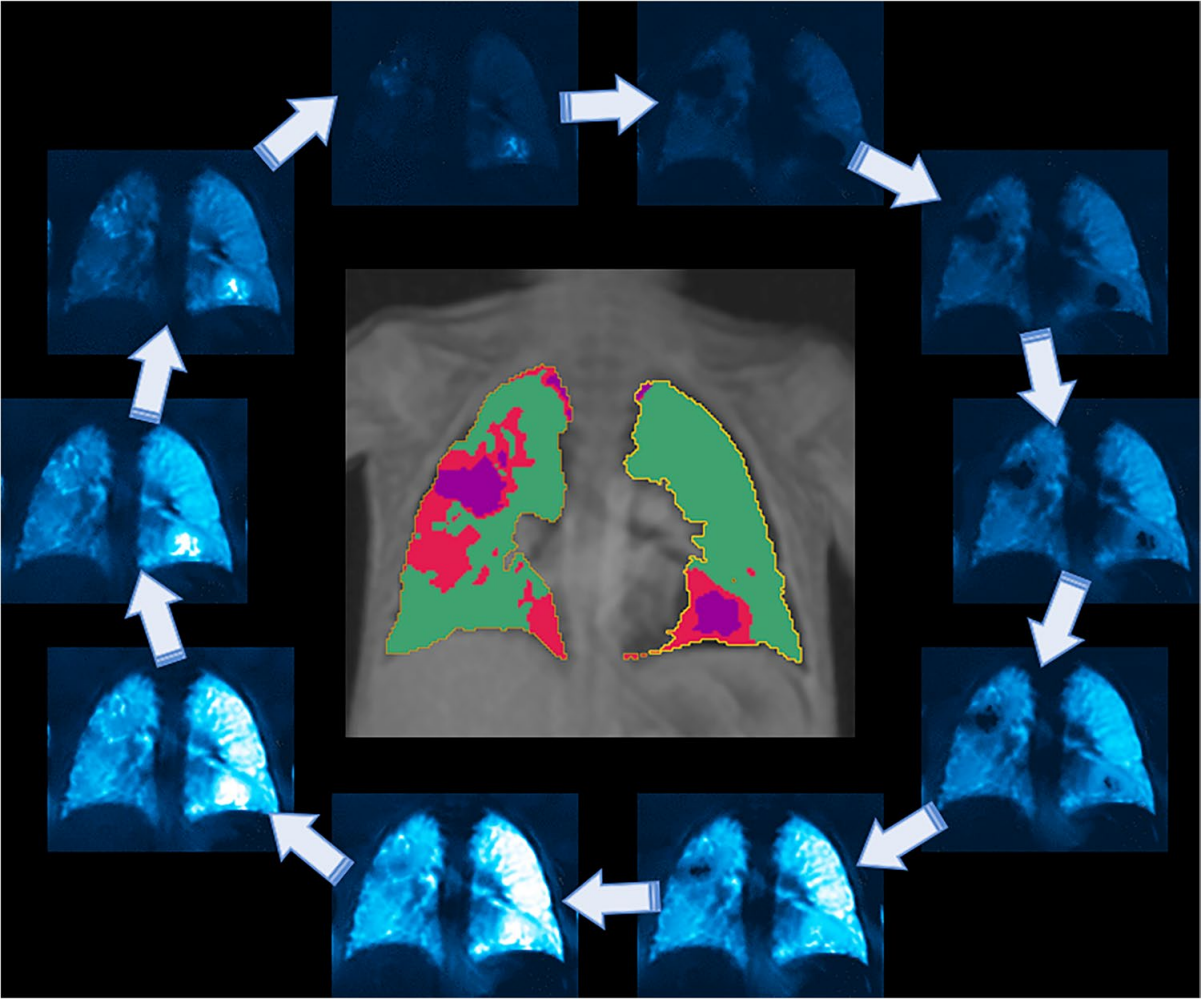
An example of an unenhanced functional proton magnetic resonance image (MRI) (Fourier decomposition) for the estimation of ventilation by means of phase-resolved functional lung MRI (PREFUL) [16] in a free-breathing 6-year-old boy after severe pneumonia (acquisition time of 60 s). Bullae can be observed in the left lower lobe (basal) and the right upper lobe, which ventilate with delayed filling and emptying of air (ventilation circle with arrows around the image). The automated computation map (within the ventilation circle) reveals healthy lung (*green*) as well as extensive damage to the lungs with perfusion deficits (*red*) and ventilation-perfusion mismatch (*purple*)

The basis for each score is the morphological, pathological alteration of the lung. These appear with different emphasis depending on the imaging modality: bronchial wall thickening, bronchiectasis, consolidations, bullae, mucus plugs and hyperinflation (Figs. 1, 2, 3, 4, 5 and 6) [16, 17]. In addition, functional assessment of regional changes in ventilation and perfusion is also desirable as complementary information.

Any change in appearance of morphological lesions forms the basis for prophylactic or therapeutic medical treatment and physiotherapy. In infective exacerbations, the imaging modality should ideally provide evidence of bacterial or mycotic aetiology. In the rare case of haemoptysis, angiographic imaging of the bronchial arteries is necessary since the haemorrhage may be treated by interventional occlusion [18].

## Why MRI?

Traditionally, imaging of the lungs has been based on conventional projection radiography. Over the past decades, multiple chest radiograph-based scores have been developed to objectify the findings in CF [19, 20]. However, a considerable amount of subjectivity persists in reporting, especially in the evaluation of conventional radiographic images. Many lesions are only visualised as nonspecific surrogate findings (e.g., ring shadows, mottled shadows and soft shadows) [21]. Artificial intelligence-based software has potential to enhance the consistency of radiographic assessment, but still requires further development [22].

Given the limitations of conventional radiography, computed tomography (CT) as a fast, high-resolution, cross-sectional diagnostic technique is, therefore commonly used by many centres for follow-up in CF [23]. In addition, unlike MRI, CT achieves sufficient quality without sedation in children younger than 5 years of age [24].

The benefits of CT notwithstanding, the risk–benefit trade-off of routine CT follow-up needs to be reevaluated [25], even when effective dose is very low [26]. As a result of expanding treatment, the number of relatively healthy children who are regularly followed up has increased. Nowadays, the transition of paediatric patients to adult pulmonology is a regular (if not routine) event. Thus, in the context of the increasing life expectancy of patients with CF, an increase in tumour disease might potentially occur among patients as a late consequence of ionising radiation [27], which should therefore be minimised.

Pulmonary MRI, the radiation-free alternative to radiographs and CT, has the disadvantage of inadequate image quality in about 10% of examinations [28]. However, with simplified respiratory gating and new sequence patterns, as described below, lung MRI examinations are increasingly simpler to perform. This is expected to drastically reduce the number of inadequate MRI lung studies. Today, lung MRI is a viable alternative to chest CT [29, 30], as reflected by the growing body of comprehensive reviews on lung MRI in patients with CF [31, 32].

## The basic protocol

Fundamental problems associated with lung imaging using MRI include the low density of protons, rapid signal loss due to T2* decay and, finally, the persistent and ubiquitous periodic motion of the diaphragm, thoracic wall, heart and great vessels. Lung MRI is feasible at both 1.5 T and 3 T [33]. Lower static magnetic field strength is beneficial for lung imaging due to its slower T2* decay [34, 35].

The basis of any pulmonary MRI is a thin-slice T2-weighted sequence [28] (Tables 1 and 2). Typically, this allows hyperintense visualisation of most pathologies: intrabronchial and alveolar mucus plugs, bronchial wall thickening, consolidations and abscesses. Given their excellent visualisation on conventional MRI sequences, these findings are also referred to as MR-plus pathologies. In contrast, smaller bullae without mass effect on surrounding lung structure and interstitial lung lesions such as fibrosis or bronchiectasis cannot reliably be detected with conventional lung sequences unless they are thick-walled or filled with mucus; thus, they are often referred to as MR-minus pathologies [36, 37]. However, this term is losing its relevance due to the more recent developments explained below [38]. T2-weighted fast spin echo sequences (FSE) with respiratory triggering are recommended for optimal quality, with an acquisition time of approximately 3 to 5 min, depending on the patient’s breathing rate. Thin slices of 3–4 mm are recommended to minimize voxel averaging that might prevent visualisation of micronodules or intralobular opacities. Often, substantially faster single-shot T2 spin echo sequences are within a few breath-hold manoeuvres; however, the clarity and hence the diagnostic confidence of these fast techniques for smaller lesions is inferior to respiratory-triggered FSE sequences [39, 40]. Therefore, even in older children, respiratory triggering for T2 imaging might be preferred. The different options for respiratory triggering (respiratory belt, diaphragm scout, phase scout in the liver or trigger systems integrated into the MR table) work comparably well and differ predominantly regarding their convenience of application [41, 42].

**Table 1:** Protocol for a 15-min, non-contrast routine morphological follow-up examination for cystic fibrosis in children younger than 6 years of age (exemplary for Siemens 3 T)

	T2 turbo spin echo	PD-UTE	T1 3-D Stack of Stars GRE (StarVIBE)
Acquisition mode	2-D	3-D	3-D
Orientation	axial/coronal	coronal	axial
Field of view (mm)	300 × 216	260	260
Matrix size (mm)	384 × 193	256 × 256	224
Slice thickness (mm)	3	3 (interpolated)	2.5 (interpolated)
Repetition time/echo time(ms)	1,000/50	3.7/0.05	3.7/2.46
Slice gap (%)	5	0	0
Flip angle (°)	120	5	9
Parallel imaging	GRAPPA 2	n/a	n/a
Respiratory compensation	triggered	self-gated	free breathing
Duration	3–5 min each	1:40 min	1:14 min

*GRAPPA* generalized autocalibrating partially parallel acquisition, *GRE* gradient echo, *n/a* not applicable, *PD* proton density, *UTE* ultra-short echo time, *VIBE* volume interpolated breath-hold examination

**Table 2:** Protocol for a 14-min, non-contrast, routine morphological follow-up examination for cystic fibrosis in children older than 8 years of age (exemplary for Siemens 3 T)

	T2 turbo spin echo	PD-UTE	T1 3-D Stack of Stars GRE (StarVIBE)
Acquisition mode	2-D	3-D	3-D
Orientation	axial/coronal	coronal	axial
Field of view (mm)	350 × 284	450	400 × 263
Matrix size (mm)	384 × 218	288	384 × 189
Slice thickness (mm)	3	2 (interpolated)	3 (interpolated)
repetition time/echo time (ms)	2,000/50	2.9/0.05	4/1.85
Slice gap (%)	5	n/a	n/a
Flip angle (°)	120	5	5
Parallel imaging	GRAPPA 2	SPIRiT 2	CAIPI 4
Resp. compensation	triggered	single breath-hold	single breath-hold
Duration	3–5 min each	0:20 min	0:20 min

*CAIPI* controlled aliasing in parallel imaging, *GRAPPA* generalized autocalibrating partially parallel acquisition, *GRE* gradient echo, *n/a* not applicable, *PD* proton density, *SPIRiT* iterative self-consistent parallel imaging, *UTE* ultra-short echo time, *VIBE* volume interpolated breath-hold examination

While T1 weighting is not mandatory for pulmonary MRI in most other conditions, it should be performed routinely in CF. The reason for this is the increased T1 signal in allergic bronchopulmonary aspergillosis, which along with the low T2 signal is considered characteristic of this disease (Fig. 5) [11]. Usually, T1 weighting is achieved as a 3-D gradient-echo acquisition in free breathing or during a single breath-hold command. Diffusion weighting may emerge as another parameter of disease severity in the future [43], but in routine diagnostics it is reserved for those situations when an abscess is suspected. Tables 1 and 2 provide exemplary protocols for different ages.

## For the ambitious

A major advance in lung MRI in recent years has been the development of ultra-short echo time (UTE) sequences (or zero-TE sequences) (Figs. 1 and 3). Extremely short echo times of less than 0.05 ms precede the rapid T2* decay in the lungs, resulting in augmented signal even from healthy lung tissue [38]. At low flip angles, a proton-density weighted impression is obtained. MR-minus pathologies particularly can be delineated more adequately with UTE sequences than with conventional MRI techniques [44]. Self-gated UTE sequences are available in free breathing where even different breathing phases can be reconstructed. In addition, highly optimised UTE sequences that allow imaging of the complete lung in a single breath-hold manoeuvre are possible. High-resolution UTE sequences are often acquired as isovoxel 3-D sequences in free breathing and with a voxel size of less than 1.5 mm, like CT, allow multiplanar reconstruction. Once manufacturers adopt these UTE sequences into their standard portfolio, they will become an integral part of routine CF imaging.

Contrast-enhanced 3-D sequences with high temporal resolution rendered by keyhole imaging techniques are widely available techniques for qualitative assessment of lung perfusion [45]. Through hypoxic pulmonary vasoconstriction, perfusion also yields indirect information about ventilation. However, in cases of haemoptysis, with bronchial artery hypertrophy as a treatable cause of bleeding, CT angiography remains the modality of choice [46]. Whether routine use of contrast-enhanced functional assessments should be recommended outside the research setting remains unclear. Acknowledging that even macrocyclic contrast agents lead to deposition in the paediatric brain [47] and considering that progressively healthier and younger patients will be given an early diagnosis and new therapies in the future, the risk–benefit of routine contrast agent administration must be questioned. Another promising new method is non-contrast perfusion, such as arterial spin labelling [48] and variants of Fourier decomposition [49]. Since the latter provides highly encouraging results [50] and is not far from transitioning into clinical practice, it will be discussed in the next section.

## For specialists

The clearest and most elaborate visualisation of ventilation is achieved with hyperpolarised gases such as ^3^helium and ^129^xenon. Thereby, a 3-D lung volume is acquired during a single breath-hold manoeuvre after the preceding inhalation of roughly 1 litre of the polarised gas. Though these methods represent ventilation disorders with high sensitivity [51, 52], three drawbacks have hindered their introduction into the clinical arena since their initial description 25 years ago: First, the sourcing and subsequent on-site polarisation of the gases is costly. Second, dedicated MR hardware enhancements are required, since standard high-frequency generators and receiver coils are designed for proton MRI [53]. Finally, handling of gas inhalation followed by immediate scanning requires extensive training. Therefore, this technique is performed only in a few specialised institutions and is not suitable for routine clinical use even in the medium term.

Fourier decomposition for the assessment of perfusion and ventilation is an important and elegant innovation in functional lung MRI and an alternative to both contrast administration and ventilation imaging with hyperpolarised gases (Fig. 6) [54, 55]. This promising technique combines perfusion and ventilation measurement and is based on ordinary proton MRI as opposed to sophisticated methods employing hyperpolarised gases. The requisite for this technique is a fast gradient echo sequence, which is available on every scanner; hence, the need for expensive investment is avoided. Thick coronal slices of about 10 mm are continuously acquired with high temporal resolution at a single slice position in free breathing. For each desired slice position, the acquisition time required for the reconstruction of one virtual ventilation and perfusion cycle is about 60 s. Subsequently, in postprocessing, ventilation and perfusion video clips as well as a quantitative mismatch map are calculated on any computer using dedicated software [56]. To date, this software is not commercially available, which means that the technology is currently only being used in a research context; however, clinical rollout is expected soon.

T1 mapping of the lung as a quantitative parameter has been proposed and explored for CF in the research context. But so far, this method has not found its way into routine clinical practice [57].

## How to report?

As a first step, an assessment of whether the quality of the specific MRI examination is sufficient or whether some minor or pronounced diagnostic limitations remain is recommended. This assessment informs the pulmonologist whether the present examination is of sufficient quality to answer all questions. In case of pronounced diagnostic limitations, a short-term repeat MRI examination or CT must be arranged, if of acute relevance. After quality assessment, a free or structured report is prepared regarding acute changes in comparison to the previous examinations focusing on findings that require immediate treatment.

The challenge of MRI reporting arises from the fact that the lesions are often multiple and very unevenly distributed in the lung and that the irregularly confined typical CF lesions are more difficult to measure than, for example, nodules. Further, the T2 signal intensity as a relative value of pathology is not readily quantifiable, although there are early encouraging results in this regard [13, 58, 59]. Attempts have been made to address these challenges with several semiquantitative CF scores for MRI [12–14]. To quantify the extent of pulmonary pathology and to reflect disease progression in a single parameter, a semiquantitative score can now be applied. It must be emphasised that CF scores, whether for CT or MRI, have a certain investigator dependence and are not well validated in terms of their predictive power for clinically relevant outcomes [60]. However, there is sufficient evidence that they correlate adequately with other surrogate parameters of disease severity, such as forced expiratory volume in 1 s [12] or the lung clearance index [10].

A simple and feasible score is the Eichinger score [13], which consists of a morphological and an independent functional (MR perfusion) component (Table 3). An extension of this score for mosaic signal intensities has been recently proposed [61]. To estimate this score, the different lobes of the lung (upper lobe, lower lobe, middle lobe and lingula) are considered separately. For each lobe, alterations of the bronchial wall, mucus plugging, abscesses, consolidation and pleural alterations are scored semiquantitatively on a scale from zero to two, depending on whether the lesion is absent in the corresponding lobe or whether it affects less or more than half of the lobe. An improvement in the quantitative assessment of hyperinflation can be expected in the future from the incorporation of ventilation inhomogeneites as shown by contrast-enhanced or unenhanced perfusion, ventilation or UTE imaging. A generic disadvantage of scores is that it is often uncertain what weights should be assigned to the individual components. This might be revolutionized by deep-learning algorithms that can independently determine prognostic criteria. First approaches to the computer-aided diagnosis of CF have already been reported for CT [62, 63]. Adapting these algorithms to MRI is complicated by the more heterogeneous image quality of lung MRI compared with chest CT, but automated quantification seems possible [59, 64].

**Table 3:** Eichinger score (modified after [13]) for morphological imaging of the lung in cystic fibrosis. Individual findings are scored according to whether pathology is absent (0), present in less than half (1) or present in more than half (2) of a lobe. If lung perfusion is recorded, an additional line can be added, which would increase the total score to 72

	R-UL	R-ML	R-LL	L-UL	L-LG	L-LL	Pathology score (total)
Bronchiectasis/wall thickening	2	2	2	2	2	2	12
Mucus plugging	2	2	2	2	2	2	12
Abscesses/sacculations	2	2	2	2	2	2	12
Consolidation	2	2	2	2	2	2	12
Pleural findings	2	2	2	2	2	2	12
Lobar score (total)	10	10	10	10	10	10	60

*L* left, *LG* lingula, *LL* lower lobe, *ML* middle lobe, *R* right, *UL* upper lobe

## What to expect for the future?

Ultra-short echo time sequences will become standard additions to conventional CF lung MRI as soon as these sequences are made widely available by manufacturers. In addition, with non-contrast perfusion and ventilation measurements of the lung, these sequences will eliminate the need for contrast administration, which is still standard in some centres due to its sensitivity.

A few fundamental disadvantages of MRI compared to CT will linger on: First, the lengthy acquisition time of MRI sequences usually requires sedation in children up to about 5 years of age. Second, there are no studies on the cost effectiveness of MRI compared to CT. However, it is likely that such a comparison will favor CT even beyond the age of infants. Finally, standardisation of MRI protocols between different manufacturers is much more challenging than for CT. Standardisation of MRI protocols and reporting is strongly advocated for the future [65] and is being advanced by the imaging group of the Clinical Trial Network of the European Cystic Fibrosis Society.

Even today, lung MRI for pulmonary monitoring in CF is performed as a preferred method in many specialised institutions [31, 32]. However, the preference for lung MRI in CF in these institutions has so far been based on individual experience. A robust assessment of advantages and disadvantages regarding sensitivity of MRI compared to CT can be anticipated from a large ongoing multicentre study in France (https://clinicaltrials.gov/ct2/show/NCT03357562).

As a full evaluation of the lung can be achieved in free breathing in about 15 min, pulmonary MRI might soon replace chest CT as a standard in CF imaging in children.
